# Household COVID-19 risk and in-person schooling

**DOI:** 10.1126/science.abh2939

**Published:** 2021-04-29

**Authors:** Justin Lessler, M. Kate Grabowski, Kyra H. Grantz, Elena Badillo-Goicoechea, C. Jessica E. Metcalf, Carly Lupton-Smith, Andrew S. Azman, Elizabeth A. Stuart

**Affiliations:** 1 Department of Epidemiology, Johns Hopkins Bloomberg School of Public Health, Baltimore, MD, USA.; 2 Department of Pathology, Johns Hopkins School of Medicine, Baltimore, MD, USA.; 3 Department of Mental Health, Johns Hopkins Bloomberg School of Public Health, Baltimore, MD, USA.; 4 Department of Ecology & Evolutionary Biology, Princeton University, Princeton, NJ, USA.; 5 Department of Biostatistics, Johns Hopkins Bloomberg School of Public Health, Baltimore, MD, USA.; 6 Institute of Global Health, Faculty of Medicine, University of Geneva, Geneva, Switzerland.; 7 Department of Health Policy and Management, Johns Hopkins Bloomberg School of Public Health, Baltimore, MD, USA.

## Abstract

In-person schooling has proved contentious and difficult to study throughout the severe acute respiratory syndrome coronavirus 2 (SARS-CoV-2) pandemic. Data from a massive online survey in the United States indicate an increased risk of COVID-19–related outcomes among respondents living with a child attending school in person. School-based mitigation measures are associated with significant reductions in risk, particularly daily symptoms screens, teacher masking, and closure of extracurricular activities. A positive association between in-person schooling and COVID-19 outcomes persists at low levels of mitigation, but when seven or more mitigation measures are reported, a significant relationship is no longer observed. Among teachers, working outside the home was associated with an increase in COVID-19–related outcomes, but this association is similar to that observed in other occupations (e.g., health care or office work). Although in-person schooling is associated with household COVID-19 risk, this risk can likely be controlled with properly implemented school-based mitigation measures.

## Back to school—safely

Severe COVID-19 in children is rare, but many schools remain closed because the transmission risk that school contact poses to adults and the wider community is unknown. Observing the heterogeneity of approaches taken among U.S. school districts, Lessler *et al.* investigated how different strategies influence COVID-19 transmission rates in the wider community using COVID-19 Symptom Survey data from Carnegie Mellon and Facebook. The authors found that when mitigation measures are in place, transmission within schools is limited and infection rates mirror that of the surrounding community.

*Science*, abh2939, this issue p. 1092

## Teaser

COVID-19 rates were elevated for those connected to schools that implemented few or no mitigation measures.

The role of schools in transmission—and the value of school closure—has been one of the most contentious issues of the COVID-19 pandemic. There is ongoing debate about exactly how much severe acute respiratory syndrome coronavirus 2 (SARS-CoV-2) risk is posed to individuals and communities by in-person schooling. Although there is general consensus that it should be possible to open schools safely with adequate mitigation measures, there are few data and even less agreement as to what level of mitigation is needed.

Many ecological studies have shown an association between in-person schooling and the speed and extent of community SARS-CoV-2 transmission (*1*–*3*), though these results have not been uniform (*4*). Although there have been numerous outbreaks in schools and school-like settings (*5*–*7*), studies outside of outbreak settings have suggested that, when mitigation measures are in place, transmission within schools is limited and infection rates mirror those of the surrounding community (*8*, *9*).

However, the ways in which in-person schooling influences community SARS-CoV-2 incidence are complex. Schools play a distinct role in the social fabric of the US and other countries and often create potential transmission connections between otherwise disparate communities. Even if transmission in classrooms is rare, activities surrounding in-person schooling, such as student pickup and drop-off, teacher interactions, and broader changes to behavior when school is in session, could lead to increases in community transmission.

There is also a growing body of evidence that younger children (e.g., those under 10 years of age) are less susceptible to infection when exposed (*10*); however, it is unclear whether they are less likely to pass on the virus once infected (*11*, *12*) or whether this reduced susceptibility is offset by increases in number of contacts during school (*13*). Even when school-aged children are infected, their risk of severe disease and death is low (*14*). This means that one of the main reasons for a focus on schools is not the risk to students, but the risk that in-person schooling poses to teachers and family members (*15*) and its impact on the overall epidemic. Yet, few studies have focused on the risk that in-person school poses to household members (*15*).

Different interpretations of the evidence and local politics have led to massive heterogeneity in approaches to schooling across the US during the 2020 to 2021 school year (*16*)—running the gambit from complete cessation of in-person learning to opening completely with no mitigation measures. Most schools that have opened have made some efforts to mitigate transmission, but there is much diversity in the approaches adopted.

This hodgepodge of approaches to schooling creates a natural experiment from which we can learn about what does and what does not work for controlling school-associated SARS-CoV-2 spread. However, there is no central repository of the measures implemented across the >130,000 schools in the US or the health outcomes in these schools. Where data are available, they are often restricted to traditional public-school systems—though 28% of prekindergarten (pre-K) through 12th-grade students are in private or charter schools—and rarely can data be linked with individual- or household-level outcomes.

The COVID-19 Symptom Survey provides an opportunity to collect and analyze data on schooling behaviors and SARS-CoV-2–related outcomes from households throughout the US. This survey is administered through Facebook in partnership with Carnegie Mellon University and yields ~500,000 survey responses in the US weekly (*17*). It includes questions on symptoms related to COVID-19, testing, and, since late November 2020, the schooling experience of any children in the household [survey details and questionnaires are available at (*18*)]. Analysis weights adjust for nonresponse and coverage bias (see materials and methods).

**Fig. 1:**
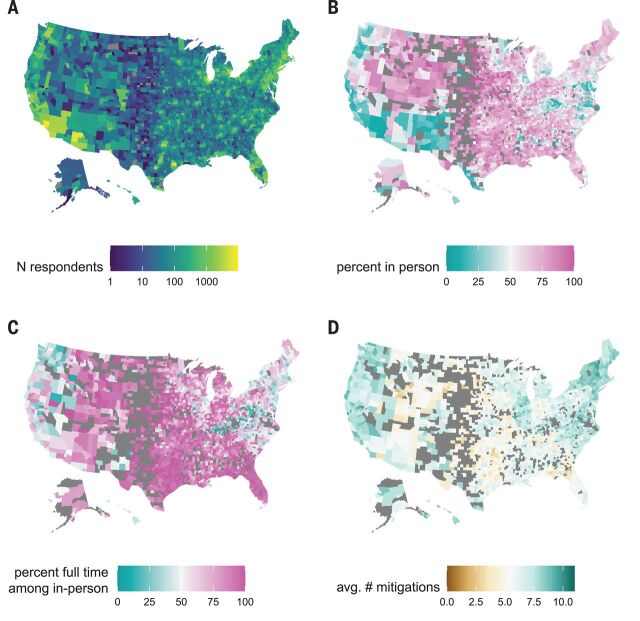
Spatial distribution of survey responses. (**A**) Number of survey respondents reporting a school-aged student in the household by county. (**B**) Percentage of households with school-aged children reporting any in-person schooling by county, excluding counties with fewer than 10 responses (excluded counties are shown in dark gray). (**C**) Percentage of households reporting a child in in-person schooling who report full-time in-person schooling, excluding counties with fewer than 10 reporting in-person schooling. (**D**) Average number of school-based mitigation measures reported for children with in-person schooling, excluding counties with fewer than 10 reporting in-person schooling.

We analyzed data collected over two time periods during the 2020 to 2021 school year (24 November 2020 to 23 December 2020 and 11 January 2021 to 10 February 2021). Of 2,142,887 total respondents in the 50 US states and Washington, DC, during this period, 576,051 (26.9%) reported at least one child in pre-K through high school living in their household (tables S1 and S2, Fig. 1A, and fig. S1). Although larger states have more responses, the per-capita response rate was fairly consistent across states (20 per 100,000; range, 10 to 29 per 100,000) and slightly higher in smaller states (fig. S2). Forty-nine percent (284,789 of 576,051) of these respondents reported a child living in the household engaged in either full- (68.8%) or part-time (46.0%) in-person schooling, with substantial variation both within and between states (Fig. 1 and table S3). Overall, in-person schooling increased between the two periods from 48 to 52%, although decreases were observed in some states (e.g., Arizona) (fig. S1 and table S3). Previous work has shown that household-reported rates of in-person schooling collected through the COVID-19 Symptom Survey track well with administrative data (*19*).

**Fig. 2:**
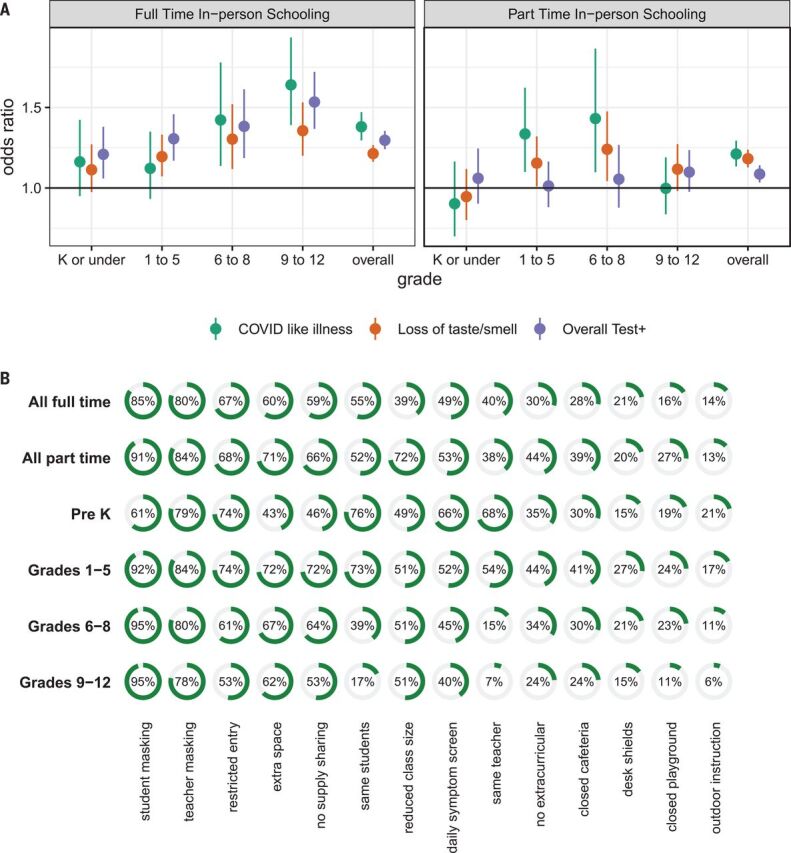
Risk from in-person schooling and distribution of mitigation measures by grade. (**A**) Odds ratio of COVID-19–related outcomes associated with full- and part-time in-person schooling by outcome and grade level compared with individuals with children in their household not attending in-person schooling and adjusted for individual- and county-level covariates (but not number of mitigation measures), which indicates that the strength of the association increases with grade level. K, kindergarten. (**B**) Distribution of mitigation measures by grade level and full- versus part-time in-person status across all grades. Test+, positive SARS-CoV-2 test result.

After adjusting for county-level incidence and other individual- and county-level factors (but not school-based mitigation measures; tables S1 and S2 and fig. S3), living in a household with a child engaged in full-time in-person schooling is associated with a substantial increase in the odds [adjusted odds ratio (aOR), 1.38; 95% confidence interval (CI), 1.30 to 1.47] of reporting COVID-19–like illness [(CLI), defined as a fever of at least 100°F, along with cough, shortness of breath, or difficulty breathing], loss of taste or smell (aOR, 1.21; 95% CI, 1.16 to 1.27), or a positive SARS-CoV-2 test result within the previous 14 days (aOR, 1.30; 95% CI, 1.24 to 1.35) (Fig. 2A and table S4). Rates of reported COVID-19 outcomes were positively correlated with county-level confirmed SARS-CoV-2 incidence (figs. S4 and S5). When stratifying by grade level (restricted to households reporting children in a single grade strata), we find that the strength of the associations with full-time schooling increases with grade (Fig. 2A and table S4).

The association between COVID-19 outcomes and reporting a child in the household engaged in part-time in-person schooling is attenuated but still statistically significant for CLI (aOR, 1.21; 95% CI, 1.13 to 1.29), loss of taste or smell (aOR, 1.18; 95% CI, 1.13 to 1.24), and reporting a positive test (aOR, 1.09; 95% CI, 1.03 to 1.14). Among those reporting part-time schooling, the association between grade and COVID-19–related outcomes is less clear (Fig. 2A and table S4).

Respondents were asked to select all mitigation measures in place for any household child engaged in in-person schooling from a list of 14 measures (see materials and methods for wording). For students engaged in any form of in-person learning, the most common mitigation measure reported was student mask mandates (88%, unweighted), followed by teacher mask mandates (80%), restricted entry (e.g., no parents or caregivers allowed into school) (66%), and extra space between desks (63%) (see table S5 for survey-weighted rates). The distribution of mitigation measures reported was similar between those reporting full- and part-time in-person schooling, though most measures were slightly more likely to be reported in the part-time setting (Fig. 2B). Besides staying with the same teacher and staying with the same students throughout the day, we found minimal evidence of clustering of mitigation measures in principal components (table S6) or hierarchical clustering analyses (fig. S6). Student mask mandates were the only intervention reported alone.

Overall, respondents reporting a household child engaged in in-person school reported a mean of 6.7 [interquartile range (IQR), 4 to 9] mitigation measures in place at any school attended. Those reporting only children in part-time schooling reported more mitigation measures (mean, 7.0; IQR, 5 to 10) than those reporting only children in full-time schooling (mean, 6.4; IQR, 4 to 9). There is substantial geographic heterogeneity in the number of mitigation measures reported (Fig. 1D, fig. S7, and tables S5 and S7), with households in South Dakota reporting the least (mean, 4.6; IQR, 2 to 7) and households in Vermont reporting the most (mean, 8.9; IQR, 8 to 11).

**Fig. 3:**
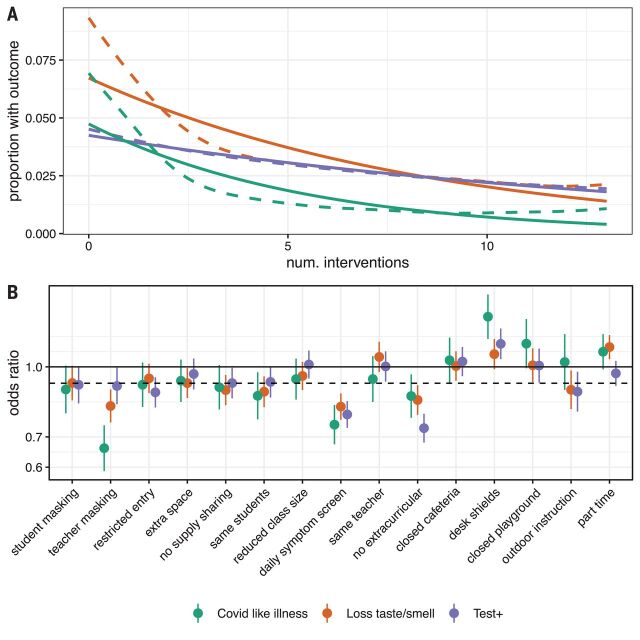
Impact of individual mitigation measures. (**A**) Relationship between number of mitigation measures and percent reporting COVID-19–related outcomes using a log-linear (solid lines) and spline (dashed lines) model. (**B**) Odds ratio of COVID-19–related outcomes by mitigation measure in multivariable model including all measures versus the reduction resulting from a generic mitigation measure (dashed line).

We find a dose-response relationship with the number of mitigation measures implemented and the risk of COVID-19 outcomes among adult household members responding to the survey after adjustment for individual- and county-level factors. On average, each measure implemented is associated with a 9% decrease in the odds of CLI (aOR, 0.91; 95% CI, 0.89 to 0.92), an 8% decrease in the odds of loss of taste or smell (aOR, 0.92; 95% CI, 0.91 to 0.93), and a 7% decrease in the odds of a recent positive SARS-CoV-2 test (aOR, 0.93; 95% CI, 0.92 to 0.94) (table S8). Regression treating each individual mitigation measure as having an independent effect shows that daily symptom screening is clearly associated with greater risk reductions than the average measure (Fig. 3 and table S9), with some evidence that teacher mask mandates and cancelling extracurricular activities are also associated with larger reductions than average. By contrast, closing cafeterias and playgrounds and the use of desk shields are associated with lower risk reductions (or even risk increases); however, this may reflect saturation effects because these are typically reported along with a high number of other measures. Notably, part-time in-person schooling is not associated with a decrease in the risk of COVID-19–related outcomes compared with full-time in-person schooling after accounting for other mitigation measures. Despite this heterogeneity in impact, we find that models including only the number of mitigation measures approximate well those where measures are modeled individually (fig. S8).

**Fig. 4:**
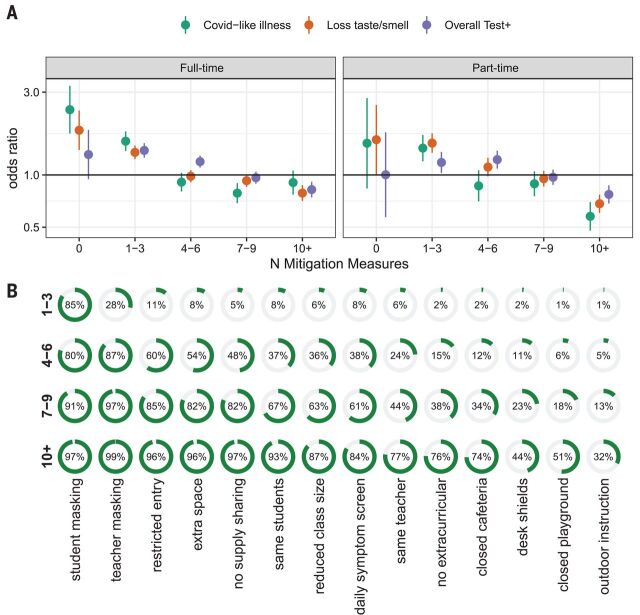
Risk of in-person schooling by strata of number of reported mitigation measures. (**A**) Estimated risk associated with full- and part-time in-person schooling by outcome and number of mitigation measures implemented, adjusted for individual- and county-level covariates. (**B**) Distribution of mitigation measures by total number of measures implemented.

To explore what, if any, levels of mitigation are associated with elimination of the risk posed by in-person schooling, we conducted analyses where the in-person exposure groups were specific to whether 0, 1 to 3, 4 to 6, 7 to 9, or 10 or more mitigation measures were reported (Fig. 4, fig. S9, and tables S10 and S11). We found that when seven or more mitigation measures were in place, the positive association between in-person schooling and COVID-19 outcomes disappeared. This result was robust to adjustment for the expected number of interventions (i.e., generalized propensity scores) on the basis of geographic- or individual-level covariates, but the result was less clear when propensity scores were based on both (fig. S10). Among those reporting seven or more mitigation measures, >80% reported student and teacher mask mandates, restricted entry, extra space between desks, and no supply sharing, and >50% reported student cohorting, reduced class size, and daily symptom screening.

The results presented here show a clear association between in-person schooling and the risk of COVID-19–related outcomes in adult household members and that this association disappears when more than seven school-based mitigation measures are reported. However, this association may not be causal, particularly given that in-person schooling and mitigation measures are not distributed randomly in the population (Fig. 1 and tables S1 to S3, S5, S7, S10, and S11). For example, households with a student attending in-person school tend to be in counties that are a higher percentage white (fig. S2) and contain respondents who are more likely to have recently eaten out or gone to a bar (table S2). Despite our best efforts to adjust for local incidence, individual behavior, and other potential confounders, it is possible that unmeasured factors drive the observed associations. Some subanalyses raise the possibility that complex interactions between geography and individual factors (but neither alone) may explain some of the observed results (fig. S10), although overadjustment is a concern in these models.

**Fig. 5:**
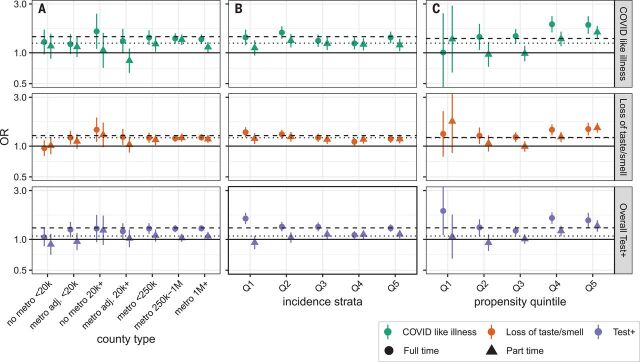
Subgroup analysis of association between in-person schooling and COVID-19–related outcomes. (**A** to **C**) Estimated odds ratios (versus those in strata not reporting in-person schooling) of COVID-19–related outcomes from full-time (circles and dashed lines) and part-time (triangles and dotted lines) in-person schooling, when data are stratified by county population size and relation to metropolitan areas (metropolitan area, nonmetropolitan area, or adjacent to metropolitan area) (A); quintile of incidence [quintile 1 (Q1) is lowest and Q5 is highest] (B); and propensity to report in-person schooling (Q5, most likely to have in-person schooling; Q1, least likely) (C). Horizontal dashed and dotted lines show overall point estimates for full-time and part-time in-person instruction, respectively.

To address the possibility that the association with in-person schooling could be the result of differences between urban, suburban, and rural counties; local patterns of incidence; or other differences between those more and less likely to send children to school in person, we performed several stratified analyses (Fig. 5). When stratifying by propensity for in-person schooling and counties classified by size and metro status, or incidence, we found few systematic or statistically significant deviations from overall estimates, even if overall rates of outcomes differed (i.e., little evidence of effect modification by strata). We found similar results when stratifying counties by reported schooling behaviors, state, percent white, poverty, and access to broadband internet (figs. S11 to S14 and table S12). The notable exception is an apparent increase in the risk associated with in-person schooling in households with a higher propensity to have children attending in-person classes (Fig. 5C).

Although we were not able to specifically examine the relationship between in-person schooling, mitigation measures, and the risk to teachers, we were able to assess the risk associated with reporting paid work outside the home among pre-K through high school teachers. Teachers working outside the home were more likely to report COVID-19–related outcomes than those working at home (e.g., test positive; aOR, 1.8; 95% CI, 1.5 to 2.2; fig. S15 and table S13). The confidence interval summarizing the elevation of risk overlapped with corresponding intervals that are associated with working in health care (aOR, 1.7; 95% CI, 1.5 to 1.9) and office work (aOR, 1.6; 95% CI, 1.5 to 1.7).

The results presented here provide evidence that in-person schooling poses a risk to those living in the households of students but that this risk can be managed through commonly implemented school-based mitigation measures. This is consistent with findings from Sweden, where authors found risk to parents and teachers using a quasi-experimental approach (*15*). However, much remains unknown. We were unable to measure the risk posed by in-person schooling to the students themselves, nor were we able to specifically assess how different policies affect teachers and other school staff. Although the interplay between school policies and local incidence is complex and, possibly, multidirectional, we find substantial variation in SARS-CoV-2 incidence regardless of the mean number of mitigation measures implemented within counties (figs. S8 and S15), and observed associations persist across study periods (figs. S17 to S19). This study also provides limited insight into the mechanisms by which in-person schooling increases risk, and it remains possible that classroom transmission plays a minor role and other school-related activities drive risk.

This study has limitations. Measures of association between COVID-19 outcomes and key exposures may be biased if confounding factors were not fully accounted for. Though we adjust for several county-level measures of socioeconomic status, these data were not available at the individual level and are known to be associated with COVID-19 risk and attitudes about in-person schooling. Analyses stratified on urbanization, background COVID-19 risk, and propensity for in-person schooling (table S5) did not reveal substantial sensitivity to the levels of factors investigated, nor did examining alternative measures of individual and household COVID-19 occurrence (figs. S20 to S22), which alleviates some of these concerns. Still, more formal studies that span schools with multiple policies and approaches would enhance insights into these questions.

Additionally, cross-sectional internet-based surveys have limitations and are subject to response biases. Although results are qualitatively consistent across COVID-19 outcomes [symptoms-based, test-based, and among those tested (figs. S20 to S22)], self-report has numerous limitations—for example, we cannot robustly assess asymptomatic spread. We were also unable to evaluate compliance with or investment in reported mitigation measures, and there is potential for mitigation measures to be reported inaccurately on the survey. Survey respondents may not be representative of the full US population, and although survey weights help account for nonresponse and coverage biases, weights calculated on the basis of the Facebook user base were adjusted for representativeness of the wider population on the basis of only age and gender—thus, these weights may not ensure representativeness across all covariates. However, the sample size of the survey and consistency of our findings across subanalyses allay some of these concerns, as does the assessment of non–COVID-19 outcomes (figs. S23 and S24). Further, any response biases would have to be differential based on schooling status to bias our results away from the null.

The debate around in-person schooling in the US has been intense and has exacerbated differences in approach between independent school systems and individual families nationally. This lack of coordination has provided an opportunity to learn about the risks of in-person schooling and the degree to which mitigation measures may reduce risk. The results presented here provide one dimension of evidence for decision makers to consider in the context of a complex policy landscape, with many competing risks and priorities. Although online surveys have their specific limitations, the wide reach of the COVID-19 Symptom Survey has allowed us to gather data from households engaged in heterogeneous schooling activities throughout the country in a way that few other studies could. In analyzing these data, we find support for the idea that in-person schooling carries with it increased COVID-19 risk to household members, but we also find also evidence that common, low-cost mitigation measures can reduce this risk.

## Supplementary Materials

science.sciencemag.org/content/372/6546/1092/suppl/DC1
Materials and Methods Figs. S1 to S26 Tables S1 to S13 References (*21*–*24*) MDAR Reproducibility Checklist Data S1 to S3

## Appendix

View/request a protocol for this paper from *Bio-protocol*.
